# Impact of Irradiated *Drosophila melanogaster* Pupae on the Quality and Population Parameters of *Trichopria drosophilae*

**DOI:** 10.3390/insects16040379

**Published:** 2025-04-02

**Authors:** Yong-Zhuo Chen, Xiao-Meng Gong, Min Zhang, Peng-Cheng Liu, Xu-Xiang Zhang, Hao-Yuan Hu

**Affiliations:** 1 School of Geography and Planning, Chizhou University, Chizhou 247100, China; 2 Collaborative Innovation Center of Recovery and Reconstruction of Degraded Ecosystems in Wanjiang Basin Co-Founded by Anhui Province and Ministry of Education, School of Ecology and Environment, Anhui Normal University, Wuhu 241000, China; 3 State Key Laboratory of Pollution Control and Resource Reuse, School of the Environment, Nanjing University, Nanjing 210023, China

**Keywords:** biological control, natural enemy, mass rearing, ^137^Cs radiation

## Abstract

*Trichopria drosophilae* is a globally distributed pupal parasitoid that targets various species within the Drosophilidae family, including the invasive *Drosophila suzukii*. The mass rearing of *T. drosophilae* is a crucial step in ensuring their successful application for field *D. suzukii* control. The pupae of *Drosophila melanogaster* are currently used as a host for the mass rearing of *T. drosophilae*. After irradiation, the immune system function of *Drosophila* pupae was weakened, leading to an increase in the parasitism efficiency of the *T. drosophilae*. Our results showed that irradiated pupae had a significant impact on the parasitism rate, offspring eclosion rate, offspring number, and female body size of F1 *T. drosophilae*, all of which were significantly lower than those in the normal group. However, there was no significant difference in the parasitism rate, body size, offspring eclosion rate, offspring number, or offspring sex ratio between F2 *T. drosophilae* emerging from treated or untreated *Drosophila* pupae. Compared with F2, F1 had a significantly higher net reproductive rate (*R₀*), mean generation time (*T*), and doubling time (*DT*), while the intrinsic rate of increase (*r*) was significantly lower. Using irradiated *D. melanogaster* pupae provides an efficient method for the mass rearing of *T. drosophilae* and offers valuable insights into its potential effectiveness in field *D. suzukii* control.

## Simple Summary

*Trichopria drosophilae* is a cosmopolitan pupal endoparasitoid of various fruit fly species, including *Drosophila suzukii.* Although many larval and pupal parasitoids have been found to effectively control *D. suzukii*, the pupal parasitoid *T. drosophilae* appears to be one of the most promising candidates, with extensive studies on the effectiveness of its parameters against *D. suzukii*. Host irradiation, which is designed to inhibit the successful development of hosts, is a standard practice in the mass rearing of various parasitoids. However, the ability of *T. drosophilae* and its offspring to successfully utilize gamma-irradiated *D. melanogaster* pupae as hosts has yet to be tested. Our results showed that irradiated pupae had a significant negative impact on the parasitism rate, offspring eclosion rate, offspring number, and female body size of F1 *T. drosophilae*, all of which were significantly lower than those in the normal group. However, there was no significant difference in the parasitism rate, body size, offspring eclosion rate, offspring number, or offspring sex ratio between F2 *T. drosophilae* emerging from the two types of *Drosophila* pupae. These results suggest that irradiated pupae influence *T. drosophilae* in the F1 generation, but the effects disappear in the F2 generation.

## 1. Introduction

*Trichopria drosophilae* Perkins (Hymenoptera: Diapriidae) is a cosmopolitan pupal endoparasitoid of various fruit fly species, including *Drosophila suzukii* Matsumura (Diptera: Drosophilidae) [1,2,3]. *Drosophila suzukii*, also known as the spotted-wing drosophila, is now widely distributed across Asia, the Americas, Europe, and Africa [4,5,6]. Female *Drosophila suzukii* are equipped with a specialized serrated ovipositor, enabling them to penetrate the epidermis of ripe, soft-skinned fruits for oviposition [7,8], which results in significant damage to fruits such as cranberries, cherries, raspberries, strawberries, grapes, blackberries, and blueberries [7,9].

The strategy of integrated pest management (IPM) for *D. suzukii* involves multiple aspects. Because the shortest egg-to-adult development period is 12 days [10], cultural management is a fundamental approach to controlling *D. suzukii*. This includes practices such as sanitation measures, timely harvesting, pruning, irrigation, mulching, and the use of exclusion netting [11]. Previous studies have shown that frequent harvesting significantly reduces the extent of fruit infestation by *D. suzukii* [12]. The common chemical method for controlling *D. suzukii* is pesticide application, which is widely adopted by fruit producers due to its simplicity and high efficiency [13]. Most broad-spectrum insecticides target the adult stage of *D. suzukii* but also impact the survival of immature stages, such as eggs and larvae [14,15]. Biological control of *D. suzukii* primarily involves the use of predators, parasitoids, and entomopathogens [5]. Although many larval and pupal parasitoids have been found to effectively control *D. suzukii* [3,16,17], the pupal parasitoid *T. drosophilae* appears to be one of the most promising candidates due to extensive studies on its efficacy against *D. suzukii* reported worldwide [2,18,19,20]. *T. drosophilae* exhibits good temperature adaptability; the parasitic efficiency of *T. drosophilae* increases as the temperature rises from 15 °C to 30 °C, leading to a shortened pre-parasitic period and lifespan. However, at 35 °C, its survival rate drops to 0%, indicating that this temperature exceeds its tolerance limit. The optimal temperature for the growth and reproduction of *T. drosophilae* is approximately 23 °C [21]. The IPM strategy of our research is to investigate the effects of irradiated pupae on *T. drosophilae*, providing a reference for the large-scale rearing of *T. drosophilae* using irradiated pupae in the future.

To effectively control *D. suzukii* through the field release of *T. drosophilae*, it is crucial to ensure that the release density of the *T. drosophilae* is sufficient to achieve significant pest suppression. In the studies conducted by Stacconi et al. (2018, 2019) [18,19] and Gonzalez-Cabrera et al. (2019) [22], *T. drosophilae* release densities of 0.3/m^2^, 1.0/m^2^, and 4.5/m^2^, respectively, were applied. All achieved effective control of *D. suzukii*, resulting in a significant reduction in its population. Previous studies have shown that *T. drosophilae* exhibits no host preference between *D. suzukii* and *D. melanogaster*, and either host can be parasitized to control *D. suzukii* [23]. Therefore, under mass-rearing conditions for *T. drosophilae*, *D. melanogaster* serves as an ideal host for *T. drosophilae* due to its higher intrinsic rate of increase, as well as its shorter generation and doubling time, which are significantly greater when parasitizing *D. melanogaster* compared with *D. suzukii* and *D. immigrans* [23].

However, parasitism under mass-rearing conditions is rarely complete, and separating parasitoids before release can be both expensive and prone to causing mechanical damage to natural enemies [24]. In addition, transporting parasitoids to field sites within their hosts, rather than as adults, is both more effective and significantly easier prior to release [25]. Host irradiation, which is designed to inhibit the successful development of hosts, is a standard practice in the mass rearing of various parasitoids [26,27,28]. The use of radiation techniques not only suppresses the emergence of non-parasitized hosts but also enhances parasitism rates and improves parasitoid fitness [28,29]. For example, irradiation of the lepidopteran hosts of the braconid parasitoid *Cotesia flavipes* (Cameron) (Hymenoptera: Braconidae) has been shown to increase parasitism rates [30]. Exposure of both *C. capitata* and *A. fraterculus* hosts to X-ray doses ranging from 20 Gy to 100 Gy resulted in increased emergence of *Diachasmimorpha longicaudata* and a female-biased sex ratio [27].

Gamma rays, X-rays, and ultraviolet-B (UVB) radiation are three methods used to prevent host emergence and enhance parasitoid production [31,32]. However, both the physiological state and body size of the host can influence the efficiency of irradiation [33,34]. Without full irradiation, maintaining large-scale parasitoid production without the risk of transporting and releasing pest flies becomes nearly impossible. Thus, gamma rays are more commonly used due to their stronger penetration ability than that of X-rays and ultraviolet-B radiation [31,35].

The aim of our research was to figure out how well *T. drosophilae* and its offspring utilize irradiated *D. melanogaster* pupae as hosts. In this study, *D. melanogaster* pupae irradiated with gamma rays were used as hosts to rear *T. drosophilae*, with evaluations conducted on the parasitism rate, emergence rate, sex ratio, longevity, and life table parameters of F_1_ and F_2_ *T. drosophilae*. Our study provides support for the efficient and convenient mass-rearing of *T. drosophilae* under laboratory conditions.

## 2. Materials and Methods

### 2.1. Insects

Laboratory populations of *D. melanogaster* and *T. drosophilae* were collected from blueberry orchards in Huangtai Village (117°52′55.56″ E, 31°20′25.48″ N), Wuhu, Anhui Province, P.R. China. All collected insects were reared in controlled environments within artificial climate chambers (25 ± 1 °C, 65 ± 5% RH, 12:12 h (L/D)). *Drosophila melanogaster* individuals were collected from Anhui Normal University, Anhui Province, China, in 2010, (31.33° N, 118.37° E) and reared in nylon cube cages (35 × 35 × 35 cm). They were fed using a 240 mL plastic cup containing 80 mL of solid food (water: 1000 mL, corn flour: 44.67 g, sucrose: 71.33 g, agar: 16.00 g, yeast: 25.00 g, propionic acid: 6.30 mL, and 100% ethanol: 22.30 mL) (Sangon Biotech, Shanghai, China). The plastic cup containing food was replaced daily, and the removed cups were kept in the climate chamber. Once the larvae were pupated on the walls of the plastic cups, the cup walls were removed and used for parasitism by *T. drosophilae*. The parasitoids were reared in plastic insect-rearing containers (10 cm in diameter, 8 cm in height) with a nylon mesh top that ensured proper ventilation while effectively preventing their escape. Fresh 2-day-old *D. melanogaster* pupae were provided to the *T. drosophilae* at a ratio of 1:20 daily for parasitism. After 24 h, the *Drosophila* pupae were removed from the rearing container and transferred to a new rearing container to develop until eclosion, where they served as the maternal generation for the next cycle of parasitoid reproduction. Additionally, cotton soaked in a 10% honey–water solution was placed inside the container to serve as a food source for the parasitoids.

### 2.2. Irradiation Treatment of Drosophila Pupae

On day 1 (24 h later), *Drosophila* pupae of a similar developmental level were carefully selected from the cup walls and placed into 50 μL transparent thin-walled tubes. To prevent damage to the pupae during collection, a small amount of sterile water was sprayed on the cup walls, and a soft brush was used to gently sweep the pupae into the thin-walled tubes. On day 2, the 2-day-old pupae of *D. melanogaster* were subjected to a 1200 Gy γ-irradiation treatment [36]. At this irradiation dose, the host’s innate immune system was not activated due to insufficient exposure, which could have led to a decrease in the parasitism rate, nor was the host’s lifespan shortened due to excessive exposure. We used the HFY-YC γ-irradiation device from the Crop and Nuclear Technology Utilization Institute, Zhejiang Academy of Agricultural Sciences, with a dose rate of 240 Gy/h.

### 2.3. Utilization of Irradiated D. melanogaster Pupae by T. drosophilae

**Figure 1:**
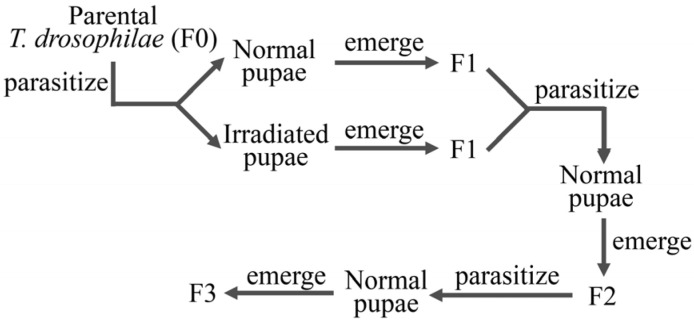
The procedure of the parasitization experiment on *T. drosophilae*.

After 24 h of parasitism by *T. drosophilae*, each *D. melanogaster* pupa was individually placed into a 50 μL transparent thin-walled tube. The eclosion of *T. drosophilae* inside the thin-walled tubes was checked daily. Newly eclosed *T. drosophilae* were selected, with 60 pairs chosen based on the principle of randomness. After mating, they were fed a 10% honey–water solution until they reached 4 days of age because the number of mature eggs of *T. drosophilae* was boosted during the first four days [37]. The female *T. drosophilae* were placed into *Drosophila* tubes (24 mm in diameter, 95 mm in height). The tubes contained cotton balls soaked in a 10% honey–water solution to provide food. Irradiated or normal 2-day-old *D. melanogaster* pupae were provided as hosts (20 pupae per group per day), with the irradiated pupae being designated as the irradiated group and the normal pupae as the normal group. After 24 h of parasitism, the female *T. drosophilae* were removed, and the parasitized pupae were individually placed into thin-walled tubes for further processing. The number and sex of eclosed *T. drosophilae* in each group were observed and recorded daily at 10:00 AM and 8:00 PM. For *Drosophila* pupae that eclosed neither into flies nor parasitoids, dissection was performed under a microscope 10 days after the first eclosion to determine their status (un-eclosed *Drosophila*, parasitized but un-eclosed parasitoids, or unknown). The eclosed *T. drosophilae* were recorded as F1 (Figure 1).

### 2.4. The Effect of Irradiated Drosophila Pupae as Hosts on T. drosophilae Reproduction

*T. drosophilae* eclosed from irradiated and normal pupae (F1) were continuously reared on normal *D. melanogaster* pupae for two generations, and life table experiments were conducted. One female and one male of F1 *T. drosophilae*, both within 12 h of eclosion, were placed into a *Drosophila* tube. The tube contained cotton soaked in 10% honey–water solution for feeding, as well as 20 normal 2-day-old *D. melanogaster* pupae as hosts. Every 24 h, fresh cotton with the honey–water solution and 20 normal 2-day-old *Drosophila* pupae were provided. After the death of the female *T. drosophilae*, no additional hosts were supplied, and only the male *T. drosophilae* were provided with cotton soaked in a honey–water solution for feeding. The parasitized pupae removed during replacement were placed in 3 mL sample tubes and kept for *T. drosophilae* eclosion. The survival status of adult *T. drosophilae*, as well as the number and sex of eclosed offspring, were observed and recorded daily. Ten days after the first eclosion, pupae that eclosed into neither *Drosophila* nor parasitoids were dissected. The *T. drosophilae* in the sample tubes were frozen at −20 °C for preservation. Afterward, their hind tibia length was measured under a microscope (VHX-5000, Keyence Corporation, Osaka, Japan).

For F0 *T. drosophilae*, we compared the parasitism rate, eclosion rate, and offspring sex ratio between the normal and irradiated groups. The normal F0 *T. drosophilae* group had 28 replicates, with each replicate consisting of 20 normal pupae per day. However, the irradiated F0 *T. drosophilae* group had 33 replicates, with each replicate consisting of 20 irradiated pupae per day. For F1 and F2 *T. drosophilae*, we compared the longevity, body size, parasitism rate, offspring eclosion rate, offspring production, and offspring sex ratio between the normal and irradiated groups. We also analyzed the effect of the increase in female *T. drosophilae* age on the number of offspring. The normal F1 *T. drosophilae* group consisted of 28 replicates, each containing 20 normal pupae per day. Similarly, the irradiated F1 *T. drosophilae* group had 26 replicates, with 20 normal pupae per replicate per day. The experimental procedure for F2 *T. drosophilae* followed the same protocol as that for F1. The normal F2 *T. drosophilae* group included 21 replicates, while the irradiated F2 group had 25 replicates, both maintaining 20 normal pupae per replicate per day.

In both the normal and irradiated groups, the longevity of F0 and F1 *T. drosophilae*, the number of offspring, the number of eclosed *D. melanogaster*, the number of *T. drosophilae* dissected, and the number of dead pupae were recorded. Based on the obtained data, four life table fertility parameters were estimated for each population: the intrinsic rate of natural increase (*r*), net reproductive rate (*R*_0_), mean generation time (*T*), and doubling time (*DT*). The value of *r* was calculated as $\sum e^{-rx}l_{x}m_{x}\;$= 1, where x was expressed as female age in days, $l_{x}$ was expressed as the age-specific survival rate, and $m_{x}$ was the number of daughters produced per female alive at age x, *R*_0_ =$\;\sum l_{x}l_{m}$, *T* = ln *R*_0_/*r*, *DT* = ln (2)/*r* [38].

### 2.5. Data Analysis

All data in this study were obtained under laboratory conditions. Groups that produced only male offspring were excluded from statistical analysis. Statistical comparisons were conducted to assess the effects of using irradiated *D. melanogaster* pupae on the parental parasitoid parasitism rate, eclosion rate, and offspring sex ratio. We analyzed the differences in lifespan, body size, parasitism rate, offspring eclosion rate, offspring sex ratio, offspring number, and population life table parameters when the *T. drosophilae* of the F1 and F2 generations parasitized irradiated and normal pupae. The eclosion rate was calculated as the number of eclosed parasitoids divided by the sum of eclosed parasitoids and parasitized but non-eclosed individuals. The parasitism rate was calculated as the sum of eclosed parasitoids and parasitized but non-eclosed individuals divided by the total number of pupae provided. We used linear fitting analysis to examine the trend of parasitism rate changes with increasing female parasitoid age. The sex ratio was equal to the number of male parasitoids divided by the total number of offspring.

For continuous data, we performed a Shapiro–Wilk normality test on longevity and body size [39]. For normally distributed data, a *t*-test was used, while the Mann–Whitney U test was applied for skewed data [40]. For binary data, the eclosion rate, parasitism rate, effect of the increase in female *T. drosophilae* age on the parasitism rate, and sex ratio were analyzed using a generalized linear model (GLM, logit link function) as the effect using a quasibinomial error due to a large residual deviance value relative to the degrees of freedom [41]. For count data, the effect of the increase in female *T. drosophilae* age on the number of offspring was analyzed using a GLM (log link function) as the effect of a quasi-Poisson error due to a large residual deviance value relative to the degrees of freedom [41]. All the tests mentioned above were performed with R version 4.2.2 [42].

## 3. Results

### 3.1. Parasitism Rate, Eclosion Rate, and Offspring Sex Ratio of F0 T. drosophilae

**Figure 2:**
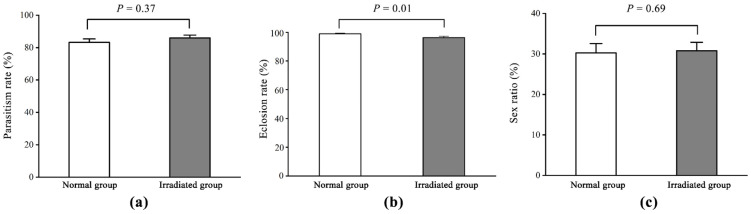
Comparison of the parasitism rate (**a**), eclosion rate (**b**), and offspring sex ratio (**c**) between the normal and irradiated groups in F0 *T. drosophilae*. Measures of variation are represented as ± SEM; *p*-values represent significant differences for the evaluated data with α = 0.05.

The parasitism rates when *T. drosophilae* used normal pupae and irradiated pupae as hosts were 83.33 ± 2.03% and 86.06 ± 1.72%, respectively, without significant differences (*F* = 0.80; d.f. = 1, 58; *p* = 0.37) (Figure 2a). The eclosion rate of normal pupae (98.90 ± 0.46%) was significantly higher than that of irradiated pupae (96.33 ± 0.88%, *F* = 6.63; d.f. = 1, 58; *p* = 0.01) (Figure 2b). The offspring sex ratio did not differ significantly between *T. drosophilae* emerging from normal (30.24 ± 2.31%) and irradiated pupae (30.80 ± 2.10%, *F* = 0.16; d.f. = 1, 58; *p* = 0.69) (Figure 2c).

### 3.2. Longevity and Body Size of F1 T. drosophilae

**Figure 3:**
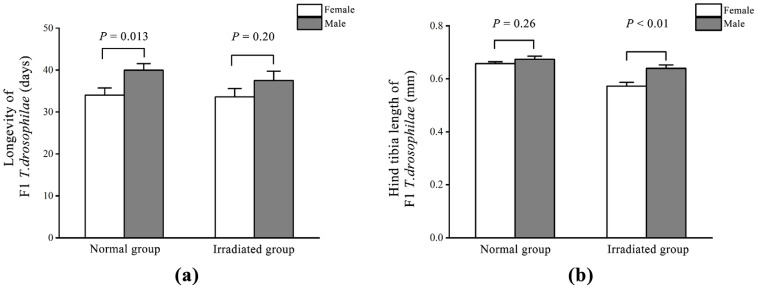
Comparison of longevity (**a**) and hind tibia length (**b**) between female and male F1 *T. drosophilae* in the normal and irradiated groups. Measures of variation are represented as ± SEM; *p*-values represent significant differences for the evaluated data with α = 0.05.

There was no significant difference in longevity between male *T. drosophilae* eclosed from normal (40.46 ± 1.57 d) and irradiated (37.50 ± 2.24 d, *t* = 0.91; d.f. = 52; *p* = 0.37) pupae. The female longevity was 34.71 ± 1.6 d in the normal group and 33.62 ± 1.98 d (*t* = 0.16; d.f. = 52; *p* = 0.87) in the irradiated group. In the normal group, the longevity of male *T. drosophilae* was significantly longer than that of female *T. drosophilae* (*t* = −2.55; d.f. = 54; *p* = 0.013) (Figure 3a). However, in the irradiated group, there was no significant difference in the longevity between male and female *T. drosophilae* (*t* = −1.30; d.f. = 50; *p* = 0.20) (Figure 3a).

As for *T. drosophilae* size, the hind tibia length of female *T. drosophilae* using normal pupae as hosts (0.66 ± 0.01 mm) was significantly longer than that of *T. drosophilae* using irradiated pupae as hosts (0.57 ± 0.01 mm, *t* = 5.54; d.f. = 52; *p* < 0.01). There was no significant difference in the hind tibia length of male *T. drosophilae* between those eclosed from normal (0.67 ± 0.01 mm) and irradiated (0.64 ± 0.01 mm, *t* = 1.88; d.f. = 52; *p* = 0.07) pupae. In the irradiated group, the hind tibia length of male *T. drosophilae* was significantly longer than that of female *T. drosophilae* (*t* = −3.38; d.f. = 50; *p* < 0.01) (Figure 3b). However, in the normal group, there was no significant difference in the hind tibia length between male and female *T. drosophilae* (*t* = −1.14; d.f. = 54; *p* = 0.26) (Figure 3b).

### 3.3. Parasitism Rate, Offspring Eclosion Rate, Offspring Production, and Offspring Sex Ratio of F1 T. drosophilae

**Figure 4:**
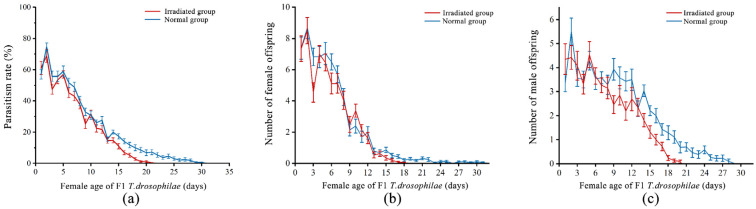
Trends in the parasitism rate (**a**), female offspring number (**b**), and male offspring number (**c**) with increasing female age in F1 *T. drosophilae* in the normal and irradiated groups. Measures of variation are represented as ± SEM.

Regardless of whether parasitoids emerged from normal or irradiated pupae, the F1 generation *T. drosophilae* achieved a maximum daily parasitism rate of 95% on normal pupae. The parasitism rate of *T. drosophilae* emerging from normal pupae (19.98 ± 0.77%) on normal pupae was significantly higher than that of *T. drosophilae* emerging from irradiated pupae (16.94 ± 0.80%, *F* = 34.83; d.f. = 1, 1847; *p* < 0.01) on normal pupae (Figure 4a). As the female *T. drosophilae* age increased, the parasitism rate significantly declined (*F* = 7033.93; d.f. = 1, 1846; *p* < 0.01) and was significantly affected by the interaction between host type and female age (*F* = 67.70; d.f. = 1, 1845; *p* < 0.01).

When F1 *T. drosophilae* parasitized normal pupae, the offspring eclosion rate was 52.54 ± 1.00% in the normal group and 42.79 ± 1.59% in the irradiated group, with the former being significantly higher than the latter (*F* = 11.39; d.f. = 1, 1007; *p* < 0.01). The offspring eclosion rate significantly decreased with increasing female F1 *T. drosophilae* age (*F* = 187.71; d.f. = 1, 1006; *p* < 0.01).

The number of offspring in the normal group (128.04 ± 3.84) was significantly higher than that in the irradiated group (108.00 ± 3.55, *F* = 32.64; d.f. = 1, 1847; *p* < 0.01). Additionally, both groups showed a decline in daily offspring production as the female age increased (*F* = 8840.46; d.f. = 1, 1846; *p* < 0.01). The interaction between host type and female age had a significant effect on offspring production (*F* = 62.27; d.f. = 1, 1845; *p* < 0.01). There was no significant difference in female offspring production between the normal (66.43 ± 2.33) and irradiated (59.54 ± 3.92, *t* = 1.53; d.f. = 52; *p* = 0.13) groups (Figure 4b). However, the male offspring production of the normal (61.61 ± 3.79) group was significantly higher than that of the irradiated (48.46 ± 4.33, *t* = 2.29; d.f. = 52; *p* = 0.03) group (Figure 4c).

**Figure 5:**
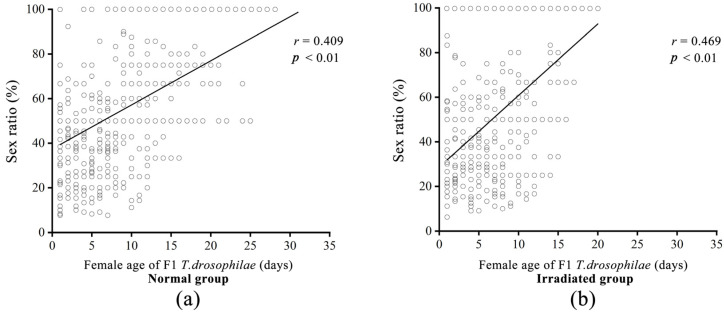
Comparison of the offspring sex ratio with increasing female age in F1 *T. drosophilae* in the normal (**a**) and irradiated (**b**) groups. The *r* value represents the Pearson correlation coefficient; the *p*-values represent statistical significance.

The offspring sex ratio was not significantly different between the normal (34.28 ± 1.24%) and irradiated (26.04 ± 1.21%, *F* = 3.67; d.f. = 1, 964; *p* = 0.06) (Figure 5a,b) groups. Increasing female F1 *T. drosophilae* age had a significant effect on the sex ratio (*F* = 257.22; d.f. = 1, 963; *p* < 0.01).

### 3.4. Life Table Parameters of F1 T. drosophilae

In the normal group, F1 female and male *T. drosophilae* emerged on days 37 and 44, respectively, with a longevity of 55 and 62 days; the age-specific survival rate $l_{x}$ declined to below 50%. The life expectancy of emerged female and male *T. drosophilae* was 34.18 days and 40.21 days, respectively. In the irradiated group, F1 female and male *T. drosophilae* emerged on days 36 and 43, respectively, with a longevity of 54 and 61 days; the age-specific survival rate $l_{x}$ declined to below 50%. The life expectancy of emerged female and male *T. drosophilae* was 33.23 days and 37.42 days, respectively.

The intrinsic rate of natural increase (*r*) was not significantly different between the normal (0.19) and irradiated (0.18, *Z* = 0.00, *p* = 1.0) groups. The net reproductive rate (*R*_0_) of the normal (66.24) group was significantly higher than that in the irradiated (59.90, *t* = 40.62; d.f. = 52; *p* < 0.01) group. The mean generation time (*T*) of the normal (22.61 d) group was significantly longer than that in the irradiated (22.47 d, *t* = 17.67; d.f. = 52; *p* < 0.01) group. The doubling time (*DT*) of the normal (3.74 d) group was significantly shorter than that in the irradiated (3.81 d, *t* = −28.72; d.f. = 52; *p* < 0.01) group.

### 3.5. Longevity and Body Size of F2 T. drosophilae

**Figure 6:**
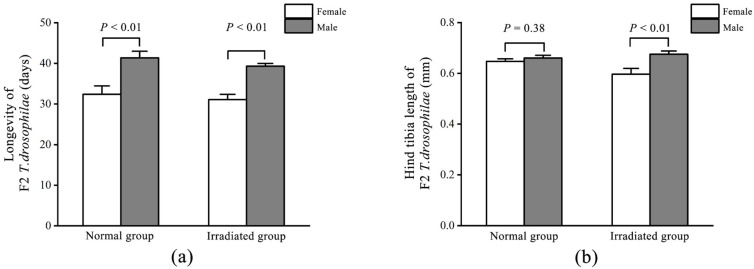
Comparison of the longevity (**a**) and hind tibia length (**b**) between female and male F2 *T. drosophilae* in the normal and irradiated groups. Measures of variation are represented as ± SEM; *p*-values represent significant differences for the evaluated data with α = 0.05.

There was no significant difference in longevity between male *T. drosophilae* eclosed from normal (41.36 ± 1.62 d) and irradiated (39.30 ± 0.70 d, *t* = 1.25; d.f. = 44; *p* = 0.22) pupae. The female longevity was 32.41 ± 2.05 d in the normal group and 31.07 ± 1.29 d (*t* = 0.57; d.f. = 44; *p* = 0.57) in the irradiated group. In the normal group, the longevity of male *T. drosophilae* was significantly longer than that of female *T. drosophilae* (*t* = −3.43; d.f. = 40; *p* < 0.01) (Figure 6a). In the irradiated group, the longevity of male *T. drosophilae* was significantly longer than that of female *T. drosophilae* (*t* = −5.62; d.f. = 48; *p* < 0.01) (Figure 6a).

As for *T. drosophilae* size, the hind tibia length of female *T. drosophilae* using normal pupae as hosts (0.65 ± 0.01 mm) was not significantly different from that of *T. drosophilae* using irradiated pupae as hosts (0.60 ± 0.02 mm, *t* = 1.63; d.f. = 44; *p* = 0.07). There was no significant difference in the hind tibia length of male *T. drosophilae* between those eclosed from normal (0.66 ± 0.01 mm) and irradiated (0.68 ± 0.01 mm, *t* = −0.86; d.f. = 44; *p* = 0.39) pupae. In the irradiated group, the hind tibia length of male *T. drosophilae* was significantly longer than that of female *T. drosophilae* (*t* = −2.95; d.f. = 48; *p* < 0.01) (Figure 6b). However, in the normal group, there was no significant difference in the hind tibia length between male and female *T. drosophilae* (*t* = −0.90; d.f. = 40; *p* = 0.38) (Figure 6b).

### 3.6. Parasitism Rate, Offspring Eclosion Rate, Offspring Production, and Offspring Sex Ratio of F2 T. drosophilae

**Figure 7:**
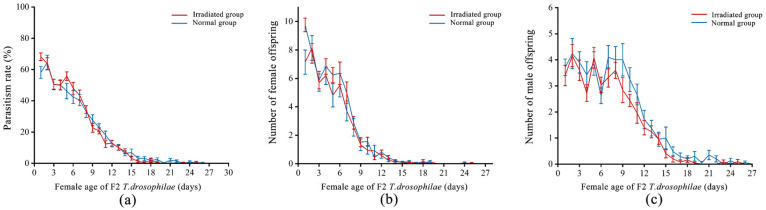
Trends in the parasitism rate (**a**), female offspring number (**b**), and male offspring number (**c**) with increasing female age in F2 *T. drosophilae* in the normal and irradiated groups. Measures of variation are represented as ± SEM.

The F2 generation *T. drosophilae* achieved a maximum daily parasitism rate of 95% on normal pupae and 90% on irradiated pupae. The parasitism rate of *T. drosophilae* emerging from normal pupae (15.13 ± 0.85%) on normal pupae was not significantly different from that of *T. drosophilae* emerging from irradiated pupae (15.80 ± 0.84%, *F* = 2.39; d.f. = 1, 1508; *p* = 0.12) on normal pupae (Figure 7a). As the female *T. drosophilae* age increased, the parasitism rate significantly declined (*F* = 2123.90; d.f. = 1, 1507; *p* < 0.01) and was significantly affected by the interaction between host type and female age (*F* = 2098.60; d.f. = 1, 1506; *p* < 0.01).

When F2 *T. drosophilae* parasitized normal pupae, the offspring eclosion rate was (41.05 ± 1.78%) in the normal group and (39.21 ± 1.64%) in the irradiated group, with no significant difference between the two groups (*F* = 0.09; d.f. = 1, 667; *p* < 0.77). The offspring eclosion rate significantly decreased with increasing female F2 *T. drosophilae* age (*F* = 45.39; d.f. = 1, 666; *p* < 0.01).

The number of offspring in the normal group (95.24 ± 5.25) was not significantly different from that in the irradiated group (95.80 ± 2.76, *F* = 1.71; d.f. = 1, 1508; *p* = 0.19). Additionally, both groups showed a decline in daily offspring production as the female age increased (*F* = 7635.93; d.f. = 1, 1507; *p* < 0.01). The interaction between host type and female age had a significant effect on offspring production (*F* = 11.00; d.f. = 1, 1506; *p* < 0.01). There was no significant difference in female offspring production between the normal (48.48 ± 3.84) and irradiated (56.00 ± 2.45, *t* = −1.70; d.f. = 44; *p* = 0.10) groups (Figure 7b). However, the male offspring production of the normal (46.76 ± 2.78) group was significantly higher than that of the irradiated (39.80 ± 2.03, *t* = 2.06; d.f. = 44; *p* = 0.05) group (Figure 7c).

**Figure 8:**
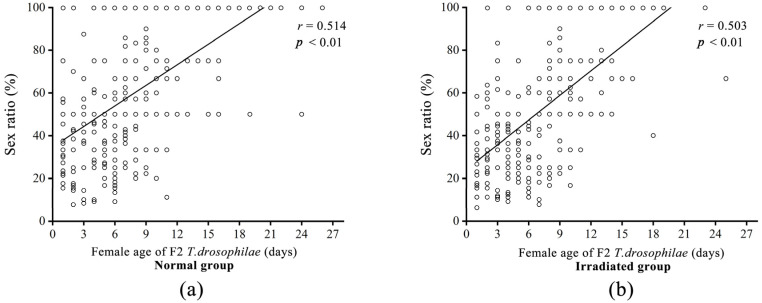
Comparison of the offspring sex ratio with increasing female age in F2 *T. drosophilae* in the normal (**a**) and irradiated (**b**) groups. *r* values represent the Pearson correlation coefficient, and *p*-values represent statistical significance.

The offspring sex ratio was not significantly different between the normal (27.21 ± 1.42%) and irradiated (22.51 ± 1.17%, *F* = 15.43; d.f. = 1, 647; *p* < 0.01) groups (Figure 8a,b). Increasing female F2 *T. drosophilae* age had a significant effect on the sex ratio (*F* = 257.22; d.f. = 1, 963; *p* <0.01).

### 3.7. Life Table Parameters of F2 T. drosophilae

In the normal group, F2 female and male *T. drosophilae* emerged on days 36 and 44, respectively, with a longevity of 53 and 61 days; the age-specific survival rate $l_{x}$ declined to below 50%. The life expectancy of emerged female and male *T. drosophilae* was 33.36 days and 42.60 days, respectively. In the irradiated group, F2 female and male *T. drosophilae* emerged on days 34 and 42, respectively, with a longevity of 51 and 59 days; the age-specific survival rate $l_{x}$ declined to below 50%. The life expectancy of emerged female and male *T. drosophilae* was 33.26 days and 39.42 days, respectively.

The intrinsic rate of natural increase (*r*) was significantly higher in the irradiated (0.19) group than in the normal (0.18, *Z* = −5.09, *p* < 0.01) group. The net reproductive rate (*R_0_*) of the irradiated (56.09) group was significantly higher than that in the normal (48.45, *t* = −40.77; d.f. = 44; *p* < 0.01) group. The mean generation time (*T*) of the normal (21.26 d) group was not significantly different from that of the irradiated (21.26 d, *t* = 0.09; d.f. = 44; *p* = 0.93) group. The doubling time (*DT*) of the normal (3.80 d) group was significantly longer than that in the irradiated (3.66 d, *t* = 24.87; d.f. = 44; *p* < 0.01) group.

### 3.8. Comparison of Quality Between F1 and F2 T. drosophilae Reared on Irradiated Pupae

The numbers of offspring produced by F1 and F2 female *T. drosophilae* were 108.00 ± 3.54 and 95.80 ± 2.76, respectively, with F1 being significantly higher than F2 (*t* = 2.70; d.f. = 49; *p* = 0.01). There was no significant difference in the offspring sex ratio between F1 and F2 (*Z* = −0.56, *p* = 0.58). Compared with F2, F1 had a significantly higher net reproductive rate (*R₀*, *t* = 19.07; d.f. = 49; *p* < 0.01), mean generation time (*T*, *t* = 146.26; d.f. = 49; *p* < 0.01), and doubling time (*DT*, *Z* = −6.20, *p* < 0.01), while the intrinsic rate of increase (*r*, *Z* = −6.28, *p* < 0.01) was significantly lower.

## 4. Discussion

Our results showed that irradiated pupae had a significant impact on the parasitism rate, offspring eclosion rate, offspring number, and female body size of F1 *T. drosophilae*, all of which were significantly lower than those in the normal group. However, there was no significant difference in the parasitism rate, body size, offspring eclosion rate, offspring number, or offspring sex ratio between F2 *T. drosophilae* emerging from the two types of *Drosophila* pupae. These results suggest that irradiated pupae influence *T. drosophilae* in the F1 generation, but the effects disappear in the F2 generation. This can be explained by the fact that irradiation can reduce host quality through the formation of reactive oxygen species (ROS) or by directly damaging macromolecules via high linear energy transfer radiation. After DNA damage occurs, the repair process initiates, leading to either cell survival or cell death [36]. One hour after irradiation, 100% mortality was observed at 1300 Gy for first-instar larvae, 1700 Gy for second-instar larvae, 1900 Gy for feeding third-instar larvae, and 2200 Gy for non-feeding third-instar larvae. This suggests that the host’s tolerance to irradiation varies across different developmental stages [36]. Additionally, this research will offer fundamental insights into the effects of using gamma-ray-irradiated hosts on parasitoid development.

Similarly to our findings, Cancino et al. (2009) [43] showed that pupae resulting from irradiated larvae were less suitable hosts than those derived from unirradiated larvae for pupal parasitoids (*Coptera haywardi* [Oglobin], *Dirhinus* sp., and *Eurytoma sivinskii*). This decline in the fitness of parasitoids parasitizing irradiated hosts may be due to the fact that insects undergoing significant developmental and metabolic changes are highly sensitive to radiation [44,45]. In addition, for koinobiont endoparasitoids, the internal physical and chemical conditions of the host must continue to provide the necessary cues and hormones to regulate the development of the parasitoid [46]. *T. drosophilae* is a specialist parasitoid that exclusively targets species within the genus *Drosophila* and exhibits exceptionally high parasitism efficiency in *Drosophila* pupae [37]. Previous research showed that after only three generations, the number of emerging offspring of *T. drosophilae* increased in both *D. suzukii* (259%) and *D. melanogaster* (35%) [47]. Therefore, we infer that F2 *T. drosophilae* is better adapted to irradiated pupae as hosts than F1 *T. drosophilae*. As a result, there is no significant difference in the offspring eclosion number between the irradiated and normal groups in the F2 generation.

In insects, a larger adult body size is typically linked to greater resource carryover from the larval stage, resulting in increased energy reserves and higher fecundity [48,49,50]. Generally, the body size of parasitoids is positively correlated with population size [51], meaning that an increase in individual body size is often accompanied by a rise in population numbers. This aligns with our findings, as the F1 *T. drosophilae* in the normal group exhibited not only larger body sizes but also higher offspring eclosion numbers compared with those in the irradiated group.

As a key factor in the effectiveness of parasitoids for biological control, maximizing the number of females enhances pest control efficiency and serves as an essential guideline for mass rearing [52,53]. Although our study found no significant differences in the sex ratio of F1 and F2 parasitoids between the normal and irradiated groups, previous research has shown that providing larger hosts can increase the number of female offspring of parasitoids. After three days of providing increasingly large hosts, *Diglyphus isaea* (Walker) sex ratios produced by groups of females dropped from 64% male to 45% male [54]. Thus, the interaction of larger hosts and radiation resulting in parasitoid feminization is crucial for optimizing the mass rearing of parasitoids for field applications.

We must clarify that all data in this study were obtained under laboratory conditions. Although *T. drosophilae* eclosion from irradiated pupae exhibited effective parasitism on normal pupae in the laboratory, environmental cues that are absent during laboratory adaptation may influence their performance in more natural settings. Few attempts have been made to use irradiated pupae as hosts to assess the parasitism performance of *T. drosophilae* in the field. Liu et al. (2023) [55] conducted 12 h outdoor quality tests and showed that approximately the same quality of parasitoids emerged from irradiated and normal pupae. Many outstanding questions remain, including how sudden changes in temperature and humidity in the field affect *T. drosophilae* parasitism and how these environmental fluctuations influence host-seeking efficiency. These are valuable topics for future research.

## Data Availability

The data that support the findings of this study were uploaded to the open repository Dryad “http://datadryad.org/share/WcZpd2zb5emcvl-cVtdxJx7TdANrveO3Bw9UMtyOs0E” (accessed on 30 March 2025).
